# Quantitative magnetic resonance spectroscopy of depression: The value of short-term metabolite changes in predicting treatment response

**DOI:** 10.3389/fnins.2022.1025882

**Published:** 2022-11-29

**Authors:** Ranchao Wang, Yu Shen, Guohai Li, Rui Du, Aiqin Peng

**Affiliations:** ^1^Department of Radiology, Affiliated Hospital of Jiangsu University, Zhenjiang, China; ^2^Department of Clinical Psychology, Zhenjiang Mental Health Center, Zhenjiang, China; ^3^Department of Radiology, Affiliated Shuyang Hospital of Xuzhou Medical University, Shuyang, China

**Keywords:** hippocampus, magnetic resonance spectroscopy, depression, prediction, treatment response

## Abstract

**Background:**

Although various prediction models of the antidepressant response have been established, the results have not been effectively applied to heterogeneous depression populations, which has seriously limited their clinical value. This study tried to build a more specific and stable model to predict treatment response in depression based on short-term changes in hippocampal metabolites.

**Materials and methods:**

Seventy-four major depressive disorder (MDD) patients and 20 healthy controls in the test set were prospectively collected and retrospectively analyzed. Subjects underwent magnetic resonance spectroscopy (MRS) once a week during 6 weeks of treatment. Hippocampal regions of interest (ROIs) were extracted by using a voxel iteration scheme combined with standard brain templates. The short-term differences in hippocampal metabolites between and within groups were screened. Then, the association between hippocampal metabolite changes and clinical response was analyzed, and a prediction model based on logistic regression was constructed. In addition, a validation set (*n* = 60) was collected from another medical center to validate the predictive abilities.

**Results:**

After 2–3 weeks of antidepressant treatment, the differences in indicators (tCho_*wee*0–2_, tCho_*wee*0–3_ and NAA _*week*0–3_) were successfully screened. Then, the predictive abilities of these three indicators were revealed in the logistic regression model, and the optimal prediction effect was found in d(tCho)_*week*0–3_-d(NAA)_*week*0–3_ (AUC = 0.841, 95%CI = 0.736-0.946). In addition, their predictive abilities were further confirmed with the validation set.

**Limitations:**

The small sample size and the need for multiple follow-ups limited the statistical ability to detect other findings.

**Conclusion:**

The predictive model in this study presented accurate prediction and strong verification effects, which may provide early guidance for adjusting the treatment regimens of depression and serve as a checkpoint at which the eventual treatment outcome can be predicted.

## Introduction

Unlike other diseases that have definitive diagnoses (Barbaresi et al., 2022; Panza and Lozupone, 2022), the mechanism of depression remains unclear (Li et al., 2022). Thus, a variety of treatment programs have been explored. Treatment response is the most important clinical indicator that determines both the sensitivity and treatment outcomes of antidepressant programs (Drysdale and Patel, 2022). In the clinical practice of depression, at least 6 weeks of observation must be taken to confirm an ineffective treatment response (Thase and Rush, 1997; Souery et al., 1999; Berlim and Turecki, 2007). Therefore, breaking the time window of 6-weeks and assessing the treatment response as early as possible will be of great clinical significance, especially for patients who may progress to refractory depression (RD). The earlier confirmation of treatment response and timely selection of another regimen (e.g., electroconvulsive therapy, magnetic stimulation, or new combination therapies) (Mitchell and Loo, 2006; Subramanian et al., 2022) may be more effective in improving their condition.

Presently, prediction is the practical solution to break the time window of treatment response. Numerous prediction studies from hospitals and laboratories have been carried out with different test or experimental methods, following a similar model: screening out the difference indicators between responding and non-responding groups at endpoints retrospectively or prospectively and predicting the treatment response at baseline (Williams et al., 2016; Emam et al., 2019; He et al., 2019). These studies can be further divided into three indicator categories: 1) gene or molecular indicators, such as inflammatory factors, VEGF (vascular endothelial growth factor), and BDNF (brain-derived neurotrophic factor); 2) tissue level indicators (mainly functional MRI), such as default mode network and functional connectivity (FC); and 3) behavioral indicators, such as childhood events and early life with parental loss or separation.

These reports have provided positive clinical and academic value for assessing and predicting depression treatment response, which also introduces two questions worth exploring. First, compared with the other two indicators (molecular and behavioral indicators), the tissue level indicator (BOLD, blood oxygenation level dependent) shows a significant advantage in non-invasiveness, convenience, and repeatability but is limited to the measurement of neural activity, which only provides indirect rather than direct indicators (Siegle et al., 2007). Second, one model showing good prediction ability in a certain sample set or laboratory may not be applicable in other depression populations that underwent different treatments or had different patient characteristics because various cofactors cannot be adequately corrected in this model based on baseline prediction. Few reliable predictors indicate which depressed individuals respond to antidepressants, as previously reported by Williams et al. (2016). For example, a history of early life trauma predicts a poorer response to antidepressant therapy, but the results are variable and limited in adults (Dunn et al., 2022). In addition to simple expansion of the sample size to improve the stability of the predictive model, the above deficiencies may also be overcome by optimizing the model.

In this study, we intended to explore the correlation between short-term metabolite changes and subsequent treatment response in depression by detecting the hippocampus (the classical treatment response region of the brain) (Huang et al., 2010) dynamically with quantitative MRS combined with a novel voxel-placement technique. Then, we built a relatively more specific and stable prediction model that provides a reference upon which to break the 6-week window of treatment response.

## Materials and methods

### Participants and procedure

**FIGURE 1:**
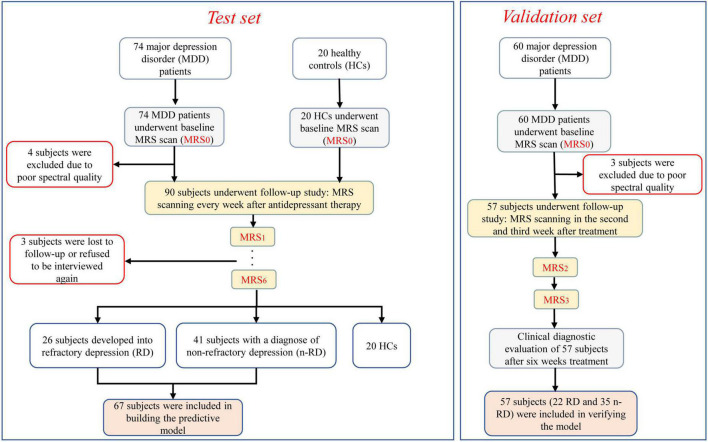
Flow chart of our study procedure. The boxes with red edge indicate excluded individuals. The light gray boxes indicate the baseline MRI scans. The yellow boxes indicate the procedure for follow-up assessments, and the light orange boxes indicate the subjects included in diagnostic model establishment and verification.

*Test set* In total, 74 major depressive disorder (MDD) patients and 20 healthy controls (HCs) were prospectively recruited from March 2017 to March 2019 at Zhenjiang Mental Health Center. All participants were examined at baseline by an experienced psychiatrist or clinical psychologist using the Screening Interview from the Structured Clinical Interview of the DSM-IV (SCID) to assess depression (Williams, 1988; Maffei et al., 1997) and met the following inclusion criteria: (1) aged 18–60 years; (2) Hamilton Depression Scale-17 (HDRS-_17_) scores of depression ≥ 17; HDRS-_17_ scores of healthy controls (HCs) < 7; (3) no general developmental disorder or mental retardation; (4) educational level above junior high school; (5) Chinese Han nationality and right-handed; and (6) voluntary participation in the 6-week follow-up. In addition, subject with any of the following condition was excluded: (1) treatment from any other psychiatric disorders; (2) pregnant or lactating female; (3) any other neurological disorders; (4) organic disorders or somatic complaints in the brain; (5)a history of alcohol or drug abuse; (6) magnetic resonance contraindications; and (7) received any treatment within 2 weeks before enrollment. After enrollment, all MDD patients were treated with citalopram for 3 weeks to observe the efficacy (*n* = 67, average dose 34.6 mg/d). Treatment responders continued to maintain the medication (*n* = 29, average dose 32.8 mg/d), and treatment non-responders were switched to the next stage for augmented treatment with bupropion (*n* = 38, average dose 237.6 mg/d) (Bech et al., 2012). During the treatment, the drug dose was increased or decreased according to the individual condition of the patients. The antidepressant effect was judged by the HAMD score reduction rate. Treatment responders were those who showed either a partial or complete response to treatment, conventionally defined as a 25–50% or >50% reduction in HAMD scores, and treatment non-responders were those who showed a < 25% reduction in HAMD scores (Drysdale et al., 2017). The subjects were then divided into RD and n-RD groups according to a HAMD score reduction rate of 50% after 6 weeks of individualized treatment. After excluding poor image quality and data of failure to follow-up, the remaining follow-up data were used for analysis. *Validation set* Another 60 depression subjects were recruited from the Affiliated Hospital of Jiangsu University from June 2019 to June 2021 for validation. The inclusion and exclusion criteria were the same as those for the test set. These subjects received corresponding treatment and underwent MRS scans at baseline and during the second and third weeks. For each patient, demographic information, past medical history and medication information were collected. The information helped the psychiatrist make decisions about individualized treatment. The study protocol was approved by the institutional ethics committee (ZJJS-2017017), and informed consent was obtained from all participants. The study protocol conformed to the ethical guidelines of the 1975 Declaration of Helsinki. Participant details and follow-up procedures are shown in Figure 1 and Table 1.

**TABLE 1:** Demographic and clinical characteristics.

		Test set MDD (*n* = 67)				Validation set MDD (*n* = 57)			
	HC (*n* = 20)	RD (*n* = 26)	n-RD (*n* = 41)	t/x^2^-value, df RD vs. n-RD	*P*-value RD vs. n-RD	RD (*n* = 22)	n-RD (*n* = 35)	t/x^2^-value, df RD vs. n-RD	*P-*value RD vs. n-RD
Age, years	30.2 ± 6.8	32 ± 8.4	28 ± 7.8	1.985, 65	0.051	34 ± 8.8	31 ± 8.6	1.271, 55	0.209
Gender, male/female	9/11	10/16	15/26	0.024, 1	0.877	8/14	13/22	0.004, 1	0.953
Education time, years	15.2 ± 2.8	14.8 ± 2.7	13.9 ± 3.1	1.216, 65	0.228	14.5 ± 3.3	13.7 ± 2.8	0.980, 55	0.331
Marital status, married/unmarried	11/9	12/14	18/23	0.033, 1	0.857	10/12	16/19	<0.001, 1	0.985
Age of onset, years	NA	21.8 ± 3.8	24.7 ± 4.1	2.901, 65	0.005	22.6 ± 4.3	25.4 ± 3.4	2.73, 55	0.009
Total duration of illness, years	NA	7.9 ± 3.2	6.2 ± 3.4	2.040, 65	0.045	8.1 ± 4.2	6.1 ± 3.7	1.886, 55	0.065
No medication (%)	20	18(69.23%)	28(68.29%)	0.007, 1	0.936	15(68.18%)	24(68.57%)	<0.001, 1	0.975
Antidepressants (%)		8(30.77%)	13(31.71%)			7(31.82%)	11(31.43%)		
SSRIs	NA	4	8	0.364, 2	0.834	4	7	0.276, 2	0.871
SNRIs	NA	2	3			2	2		
NaSSAs	NA	2	2			1	2		
HDRS_–17 (week0)_	5.5 ± 1.2	25.2 ± 4.1	24.6 ± 3.8	0.611	0.543	25.7 ± 3.7	24.8 ± 3.5	0.925	0.359
HDRS_–17 (week6)_	5.4 ± 1.3	18.3 ± 5.4	7.6 ± 3.7	9.631	<0.001	16.8 ± 4.8	6.8 ± 3.2	9.450	<0.001

MDD, major depression disorder; HC, healthy control; RD, refractory depression; n-RD, non-refractory depression; HDRS-17, 17-item Hamilton Rating Scale for Depression; Antidepressants are taken at least 3 months ago; SSRIs, selective serotonin reuptake inhibitors; SNRIs, serotonin–norepinephrine reuptake inhibitors; NaSSAs, noradrenergic and specific serotonergic antidepressant; df, degree of freedom; Data are shown as mean ± SD; NA, not available.

### MRI acquisition

MRI acquisitions were performed on a Siemens 3.0T Trio MR scanner using an eight-channel head coil. Subjects were instructed to keep their eyes closed, relax, remain immobile, think of nothing in particular, and avoid falling asleep. Initial image acquisition included a T1-weighted image scan acquired with a Magnetization Prepared Rapid Gradient Echo (MPRAGE) sequence (TR/TE/flip angle = 2,530 ms/2.26 ms/90°; voxel size = 1 × 1 × 1 mm; FOV = 256 × 256 mm^2^; matrix = 256 × 256; slice thickness = 1 mm). Additional sequences (such as T2 and FLAIR) were performed to ensure that all studied participants were MRI-negative (no imaging findings of organic lesions). Axial and coronal images were reconstructed based on the sagittal images for MRS localization.

**FIGURE 2:**
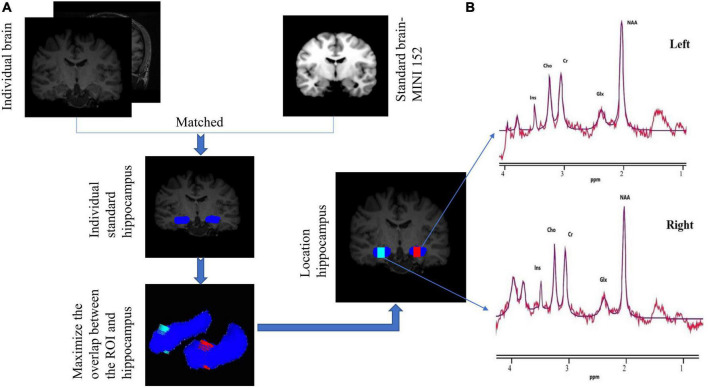
**(A)** Flow chart of the MRI data processing procedure. The blue part represents the bilateral hippocampus of the individual. The voxel box has the largest overlap rate with the left hippocampus (red) and the right hippocampus (green). Coronal individual images showing the size (in voxels) and the location of the left (red) and right (green) hippocampus. **(B)** 1H-MRS obtained from the voxels at the individual level (red line) and the overlay of the spectral fit (purple line). All spectral data are analyzed with jMRUI version 5.2. NAA, N-acetyl-aspartate; Cho, phosphocholine and glycerophosphocholine; Cr, creatine and phosphocreatine; Glx, glutamate and glutamine; Ins, myo-inositol; ppm, parts per million.

Firstly, the brain template (Montreal Neurological Institute) was registered to the subject’s DICOM images to acquire the transformation parameters (Dou et al., 2015). Simultaneously, the subject’s standard hippocampus was extracted with the FMRIB software library (FSL)^1^ and the ROI (the region of interest) mask was created (size: 10 × 10 × 15 mm^3^). Secondly, the in-house developed software was designed to achieve maximum overlap between ROI and hippocampus. The in-house developed software was based on the iterative algorithm procedure, formulated in MATLAB (MATLAB 2015b; Mathworks, Natick, MA) and described as: 1.the overlapping volume of ROI placement and hippocampus area was calculated; 2. the spatial position of the ROI was constantly adjusted, and its overlapping volume was recorded; and 3. the position with the largest overlapping volume in all calculation results was compared and determined. Then, Voxel’s coordinates, size, angulation about each axis, image orientation (“LAS”) were recorded in “Voxel_ Location. Txt” file for rapid and accurate voxel placement of future follow-up (Woodcock et al., 2018). Thirdly, the transformation parameters were used to acquire the subject’s voxel placement mapped by the voxel mask in atlas space (Figure 2A). The voxel overlap was defined as the percentage of the week 0 voxel volume encompassed by the follow-up voxel. The geometric voxel overlap suggested high accuracy of voxel placement across all subjects (mean overlap of each subject’s voxel = 93.4% ± 4.3% during the 6-week follow-up).

Single-voxel spectra (SVS) were acquired by using a standard point resolved spectroscopy (PRESS) sequence. Water-suppressed SVS was performed with VAPOR water suppression and the following parameters: echo time (TE) of 35 ms, repetition time (TR) of 2,000 ms, nominal voxel size: 10 × 10 × 15 mm^3^, spectral width of 5,000 Hz, 2048 time points, and 128 averages. Spatial saturation pulses were applied to minimize contamination of the signal from outside the voxel. Linear shims were used to correct the B0 inhomogeneity across the investigated voxel. Water MR spectroscopic spectra were also acquired without water suppression on the same voxel, with TE = 20 ms and all other parameters remaining the same. The acquisition in the oblique coronal plane was perpendicular to the long axis of the hippocampus.

### Magnetic resonance spectroscopy data processing

The spectra were pre-processed (including phased and apodized 1 Hz) (Tiwari et al., 2020; Near et al., 2021) and then analyzed with software jMRUI (version 5.2). The time-domain quantification of metabolite signals was conducted using *AMARES* algorithm with custom prior knowledge. Metabolite concentrations were reported for tCr (creatine plus phosphocreatine), NAA (N-acetyl-aspartate), tCho (phosphocholine and glycerophosphocholine), Ins (myo-inositol) and Glx (glutamate and glutamine). The AMARES prior knowledge model consisted of peaks for NAA, choline (Cho), creatine (Cr), glutamate + glutamine (Glx) and myo-inositol (Ins). The amplitudes of NAA, Cho, Cr, Glx, and Ins peak were estimated by the algorithm. The relative phases of NAA, Cho, Cr, Glx, and Ins peak were fixed at 0. The linewidth of NAA was estimated by the algorithm, and the linewidths of the remaining peaks were set to be equal to that of NAA. The frequencies of NAA, Cho, Cr, Glx, and Ins peak were estimated by AMARES. All peak shapes were fixed at Lorentzian. Data were subjected to quality control prior to inclusion in the analysis. We required spectra to meet the following criteria to be eligible for inclusion: the signal-to-noise ratio (SNR) ≥ 15, the full width at half maximum (FWHM) ≤ 16 Hz, and the Cramér-Rao lower bounds (CRLBs) < 20% (Supplementary Table 3). The spectral analysis window was defined as 0–4.0 ppm (Figure 2B). Absolute concentrations of metabolites were calculated using water signal from the identical voxel as internal reference. The relaxation times (T1 and T2) of water and respective metabolites measured at 3 T were used for relaxation correction. The concentrations were calculated according to Kreis et al. (Kreis et al., 1997) as follows: C_*M*_ = (S_*M*_/S_*W*_) × C_*W*_ × (n_*W*_/n_*M*_) × (f_*W*_^*T*1^/f_*M*_^*T*1^) × (f_*W*_^*T*2^/f_*M*_^*T*2^), where indexes M for metabolite and W for water, C stands for concentration, S for signal intensity, n for the number of chemically equivalent protons (contributing to the signal), f^*T*1^ for spin-lattice relaxation function (1-e^*TR/T*1^), f^*T*2^ for spin-spin relaxation function (e^–TR/T2^). C_*W*_ stands for concentration of water in white matter, which is 55.51 moles/kg.^2^ To correct the metabolites’ concentration of cerebrospinal fluid (CSF) contamination, the CSF, gray matter (GM) and white matter (WM) volumes were segmented and calculated from T1-weighted images by FSL. The calibration formula (Near et al., 2021) was as follows: C_*cor*_ = C_*raw*_ × [V_*total*_/V_*total*_-V_*CSF*_)], where C_*cor*_ denoted the corrected value; C_*raw*_, the uncorrected value; V_*total*_, the voxel volume; V_*CSF*_, the CSF volume (Supplementary Table 4). In brief, voxel tissue composition and unsuppressed endogenous water were used to calculate and calibrate absolute concentrations of metabolites. The mean metabolite concentration of bilateral ROI was used in analysis. Test-retest reliability analysis gave ICC of 0.78, 0.75, 0.88, and 0.83 (NAA, Cr, Cho, and Ins) in healthy control group according to the metabolic measurements of week 0 and week 1, indicating excellent reliability, while the Glx (0.64) was not.

### Statistical analysis

Statistical analyses were performed using GraphPad Prism 7 (GraphPad Software Inc., La Jolla, CA) and R 3.1.2 software (R Foundation for Statistical Computing, Vienna, Austria). All analyses were two tailed with an alpha level of 5%. Data were tested for normality (Shapiro--Wilk test) and homogeneity (Levene’s test). Clinical and demographic data are presented as the means and SD, with appropriate tests for intergroup comparison (*t*-test for continuous data and X^2^ for categorical data). Test-retest reliability was calculated in healthy control group based on metabolites concentrations including NAA, Cr, Cho, Ins and Glx in week 0 and week 1, which was assessed with the intraclass correlation coefficient (ICC). The ICC values > 0.7 indicated good reliability. Trend analyses between groups were performed using ANOVA to detect whether the metabolite levels changed with increasing treatment time. The intragroup [RD_*week(n)*_ vs. RD_*baseline*_, n-RD_*week(n)*_ vs. n-RD_*baseline*_) and intergroup (RD_*week(n)*_ vs. n-RD_*week(n*)_] differences in hippocampal metabolite concentrations were assessed by *t*-test. All differences were further verified by a general linear model (GLM) correcting covariates (age, gender, age of onset, total duration of illness). All differentiated changes were defined as the week of follow-up minus the week of baseline. The relationship between the changed HDRS scores and changed metabolite concentrations in the short term was assessed using Pearson’s correlation coefficients. Stepwise logistic regression was used to screen the changed hippocampal metabolites with predictive capability in the short term to discriminate RD and n-RD with correcting covariates (age, gender, age of onset, total duration of illness). Then, the receiver operating characteristic (ROC) curve was used to analyze the performance of predictors in terms of AUC (area under curve), sensitivity and specificity. The statistical methods used in the validation cohort were consistent with those used in the test cohort.

## Results

### Longitudinal evaluations of hippocampal metabolites in week 0–week 6

**FIGURE 3:**
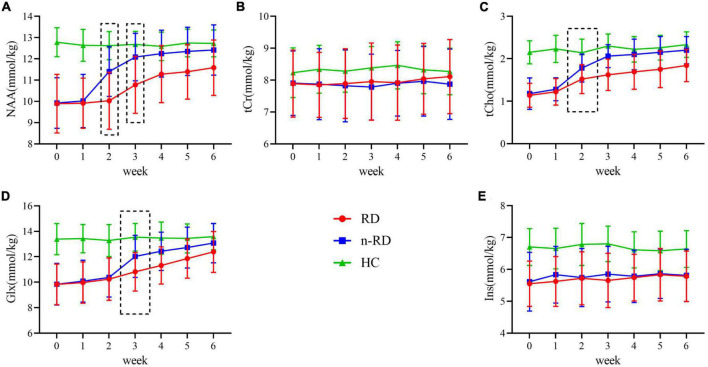
**(A–E)** Longitudinal concentration change of metabolites over the follow-up period. The mean concentrations of metabolites in hippocampus for all subjects was divided into the following categories according to clinical diagnosis: healthy controls (HC/green, *n* = 20), refractory depression group (RD/red, *n* = 26) and non-refractory depression group (n-RD/blue, *n* = 41). NAA, N-acetyl-aspartate; tCho, phosphocholine and glycerophosphocholine; Glx, glutamate and glutamine; tCr, creatine and phosphocreatine; Ins, myo-inositol.

Beginning in the second week, the tCho concentration showed a gradual increase both in the RD (p for trend < 0.001; t_*week*0–2_ = 4.40, *p* < 0.001) and n-RD groups (p for trend < 0.001; t_*week*0–2_ = 7.75, *p* < 0.001). The Glx concentration showed a similar trend beginning in the third week (RD: p for trend < 0.001; t_*week*0–3_ = 2.28, *p* = 0.027; n-RD: p for trend < 0.001; t_*week*0–3_ = 6.02, *p* < 0.001). However, there was a slight difference in the trend of NAA concentration between RD and n-RD; the former increased after the third week (p for trend < 0.001; t_*week*0–3_ = 2.36, *p* = 0.022), while the latter increased after the second week (p for trend < 0.001; tweek_0–2_ = 4.52, *p* < 0.001). Furthermore, there was no significant longitudinal trend in Ins and tCr (all p for trend > 0.05), which indicated that they might be unrelated to the treatment response (Figure 3). Detailed data are shown in Supplementary Material 1.

### Metabolite changes after short-term treatment

**FIGURE 4:**
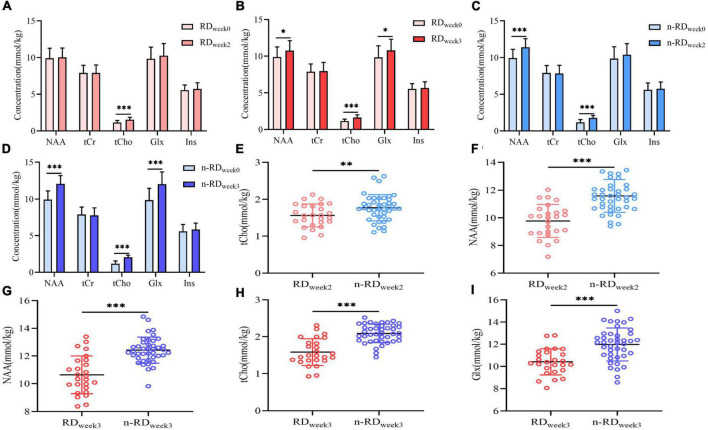
**(A–D)** The within-group comparisons of longitudinal metabolic change in refractory depression group and non-refractory depression group show significant differences after 2 and 3 weeks of treatment. **(E–I)** The between-group comparisons of metabolic change in refractory depression group and non-refractory depression group show significant differences after 2 and 3 weeks of treatment. NAA, N-acetyl-aspartate; tCho, phosphocholine and glycerophosphocholine; tCr, creatine and phosphocreatine; Glx, glutamate and glutamine; Ins, myo-inositol; *p < 0.05, **p < 0.01, ***p < 0.001.

Given the above results, indicators of tCho, Glx, and NAA were used for further observation of the treatment response. In the intragroup comparison, after two weeks of antidepressant treatment (week 0-week 2), the concentration of tCho significantly increased in both the RD and n-RD groups, and the NAA concentration only increased in the n-RD group (Figures 4A,C and Supplementary Table 1). After three weeks (week 0–week 3), significant increases in NAA, tCho, and Glx concentrations were found in both the RD and n-RD groups (Figures 4B,D and Supplementary Table 1). In the intergroup comparison, compared with the RD group, the concentrations of tCho and NAA in the n-RD group were significantly higher at the second week (tCho *t* = 3.11, *p* = 0.003; NAA *t* = 4.53, *p* < 0.001) (Figures 4E,F), and the tCho, NAA and Glx concentrations were significantly higher at the third week (tCho *t* = 5.62, *p* < 0.001; NAA *t* = 4.29, *p* < 0.001; Glx *t* = 3.02, *p* = 0.004) (Figures 4G–I).

### Correlation between metabolite concentration and clinical response

**FIGURE 5:**
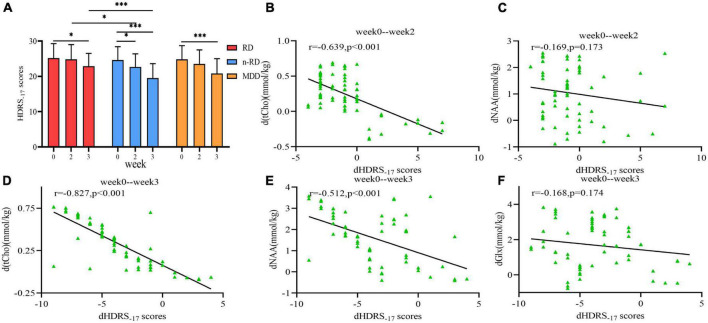
**(A)** The within-group and between-group comparisons of Hamilton Rating Scale scores change in refractory depression group, non-refractory depression group and major depression disorder group show significant differences. **(B–F)** Scatter plots depict the relationship between metabolites changes (d(tCho), dNAA and dGlx) in hippocampus and the clinical treatment response (dHDRS) after 2 and 3 weeks. HDRS-17, Hamilton Rating Scale 17-item for depression; NAA, N-acetyl-aspartate; tCho, phosphocholine and glycerophosphocholine; Glx, glutamate and glutamine; *p < 0.05, **p < 0.01, ***p < 0.001.

Improved HDRS scores of MDD were found until the third week (t_*week*0–3_ = 5.71, *p* < 0.001), in which a definite decrease in HRDS scores was found in the n-RD group since the second week (t_*week*0–2_ = 2.15, *p* = 0.037) and the RD group since the third week (t_*week*0–3_ = 2.29, *p* = 0.024) (Figure 5A). Subsequent correlation analysis showed that differentiated HDRS (dHDRS) was in negatively correlated with differentiated tCho [d(tCho)] (*r* = −0.639, *p* < 0.001) but not with differentiated NAA (dNAA) (*r* = −0.169, *p* = 0.173) in week 0-week 2 (Figures 5B,C), and dHDRS was negatively correlated with d(tCho) (*r* = −0.827, *p* < 0.001) and dNAA (*r* = −0.512, *p* < 0.001), but not with differentiated Glx (dGlx)(*r* = −0.168, *p* = 0.174) in week 0-week 3 (Figures 5D–F).

### Prediction of treatment response

**FIGURE 6:**
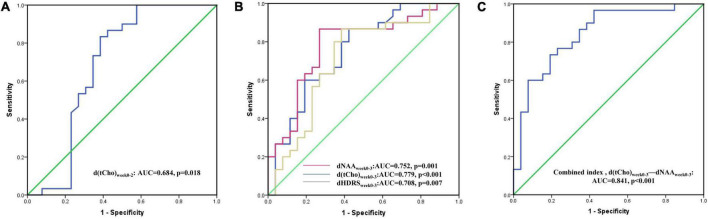
**(A–C)** ROC curves of hippocampal metabolites predictors in the second and third week are presented. ROC, receiver operating characteristic. AUC, area under curve; NAA, N-acetyl-aspartate; tCho, phosphocholine and glycerophosphocholine; HDRS, Hamilton Rating Scale for Depression.

Based on the results of the correlation analysis, dHDRS_*week*0–2_ + d(tCho)_*week*0–2_ and dHDRS_*week*0–3_ + d(tCho)_*week*0–3_ + dNAA_*week*0–week3_ were included in further stepwise logistic regression analysis with other factors (age, gender, age of onset, total duration of illness). The results showed that d(tCho)_*week*0–2_ was an independent predictor for treatment response at the second week (OR = 0.429, *p* = 0.01; AUC = 0.684), but dHDRS_*week*0–2_ was not (OR = 1.495, *p* = 0.371). At the third week, although dHDRS_*week*0–3_ presented a non-neglectable predictive value (OR = 3.179, *p* = 0.041; AUC = 0.708), better predictive capabilities were found with d(tCho)_*week*0–3_ (OR = 0.115, *p* < 0.001; AUC = 0.779) and dNAA_*week*0–3_ (OR = 0.117, *p* = 0.033; AUC = 0.752). Furthermore, improved capability was obtained with the combined index [d(tCho)_*week*0–3_ and dNAA_*week*0–3_] (AUC = 0.841, *p* < 0.001) (Figure 6 and Table 2).

**TABLE 2:** Logistic regression analysis in check point week 2 and week 3.

Variables	β	S.E.	Wald	*P*	OR (95% CI)
dHDRS_week0–2_	0.402	0.450	0.798	0.371	1.495 (0.619∼3.614)
d(tCho) _week0–2_	−0.846	0.328	6.704	0.01	0.429 (0.251∼0.735)
d(tCho) _week0–3_	−2.167	0.352	37.897	<0.001	0.115 (0.057∼0.228)
dNAA_week0–3_	−2.145	1.002	4.582	0.033	0.117 (0.024∼0.608)
dHDRS_week0–3_	1.156	0.544	3.672	0.041	3.179 (1.836∼5.501)

d(tCho)_week0–2_, differentiated tCho (phosphocholine and glycerophosphocholine) after two weeks of treatment; d(tCho)_week0–3_, differentiated tCho after three weeks of treatment; dNAA_week0–3_, differentiated NAA (N-acetyl-aspartate) after three weeks of treatment; dHDRS _week0–2_, differentiated HDRS (Hamilton Depression Rating Scale) scores after 2 weeks of treatment; dHDRS_week0–3_, differentiated HDRS scores after 3 weeks of treatment.

### Model validation

In the validation set of 57 subjects (22 RD and 35 n-RD patients), good diagnostic value was obtained with d(tCho)_week0–2_ (accuracy = 68.42%, AUC = 0.708) after two weeks of treatment, and better performance was found in d(tCho)_week0–3_ (accuracy = 75.44%, AUC = 0.785), dNAA_week0–3_ (accuracy = 71.93%, AUC = 0.722) and d(tCho)_week0–3_-dNAA_week0–3_ (accuracy = 85.96%, AUC = 0.837) after 3 weeks of treatment (Table 3). Detailed data are shown in Supplementary Table 2.

**TABLE 3:** Diagnosis accuracy of the MRS metabolites predictors.

Actual	Predictor							
	d(tCho)_week0–2_		d(tCho)_week0–3_		dNAA_week0–3_		d(tCho)_week0–3_-dNAA_week0–3_	
	RD	n-RD	RD	n-RD	RD	n-RD	RD	n-RD
RD	14	8	16	6	16	6	18	4
n-RD	10	25	8	27	10	25	4	31
Accuracy (%)	68.42		75.44		71.93		85.96	

RD, refractory depression; n-RD, non-refractory depression; d(tCho)_week0–2_, differentiated tCho (phosphocholine and glycerophosphocholine) after 2 weeks of treatment; d(tCho)_week0–3_, differentiated tCho after three weeks of treatment; dNAA_week0–3_, differentiated NAA (N-acetyl-aspartate) after three weeks of treatment; d(tCho)_week0–3_-dNAA_week0–3_, the combined detection of dNAA and d(tCho) after 3 weeks of treatment.

## Discussion

In this study, combining quantitative MRS with a new method of ROI positioning, we conducted a longitudinal follow-up from baseline to 6 weeks in MDD and constructed an improved prediction model, in which several interesting findings were reported: (i) After 2 weeks of treatment, the changed tCho concentration could accurately predict the subsequent treatment response, but the changed HDRS score could not. (ii) After 3 weeks of treatment, although the changed HDRS score could predict treatment response, indicators from MRS (changed tCho and NAA) showed a stronger predictive power. (iii)The new ROI positioning strategy and predictive model presented a more stable verification capability.

Previous studies have indicated that hippocampal metabolites at baseline are positive predictors that suggest functional conditions, which may dominate the treatment responses (Block et al., 2009). In fact, this predictive model was an ideal model based on standard conditions, in which the individual differences, disease status and treatment plans were ignored, which might be why the predictive results of different laboratories could not be unified or well verified. In our relatively conservative predictive model, the predicted time point was placed 2 or 3 weeks after short-term treatment. In other words, the change in indicators from week 0 to week 2 or week 3 was used to predict the outcome of treatment response, and our results also yielded a positive verification effect in the validation group. In brief, the predictive model of this study was reported for the first time in imaging research on depression, which brings the novelty to the field.

In addition to the joint application of programming language and FSL segmentation, this study also applied a novel voxel-placement technique. The realization of ROI localization with accurate overlap in multiple scans is the key and difficult point of MRS studies. The perfect match was considered impossible by hand sketching in the existing studies, especially in the irregular and small gray matter nuclei such as the hippocampus and amygdala. Compared with other existed approaches, the highlight of our technique was the maximum overlap region which was acquired by iterating the ROI and the individual standard hippocampus. We meant to make the voxel include as much hippocampus tissue and little contamination from surrounding tissue as possible, which could make it more representative. Other approaches were developed to improving voxel placement technique for reliable voxel coregistration within- and between-subjects, and we had benefited from these approaches. For example, after the maximum overlap region between ROI and hippocampus was determined, we also used the transformation parameters which were calculated from the registration of the atlas to the skull-stripped subject’s brain to acquire the subject’s voxel placement mapped by the voxel mask in atlas space (Park et al., 2018). And we also created the file which includes specific individual voxel information to ensure high precision division, maximum overlap and high repeatability in future follow-up (Woodcock et al., 2018). Besides, our technique has a similarity that the brain template space was used for registration. However, other research (Hancu et al., 2005; Dou et al., 2015) used the registration technique based on the first scan session. This precise and stable voxel placement method might deserve further improvement and promotion in sensitive quantitative single-voxel NMR spectroscopy.

In general, studies on hippocampal choline levels and their changes during treatment have given conflicting findings. Some studies have reported increased choline levels at baseline (Milne et al., 2009), while others have shown different results: major depressive disorder (MDD) patients with first episodes had a trend toward lower choline levels, and those with remitted recurrence had higher choline levels than controls (de Diego-Adeliño et al., 2013). MDD patients with a first episode accounted for a large proportion of the subjects in our study. In addition, depression is a heterogeneous disease in which different patients might have significant individual differences (manifested in symptoms, treatment differences, etc.), which might impede the consistency of choline level reports. Above all, the contribution of surrounding tissue (non-hippocampal region in voxel) to choline levels remains unclear. We optimized the placement of voxels in the hippocampus to include more hippocampal tissue and less surrounding tissue to make it more representative, while the quantitative region centered on the hippocampus contained more contributions from surrounding tissues based on traditional voxel placement (Milne et al., 2009). These may be the reasons why the choline level in MDD patients reported by us was lower than that in some other studies. Some studies have reported no change in choline levels during treatment (Wang et al., 2012). However, others have reported that the NAA and choline increase in the hippocampus in association with pharmacological treatment response and that these changes are applicable particularly for patients with low NAA and Cho baseline levels (Block et al., 2009). The different choline levels and their change in treatment reported by different studies may implicate different pathophysiological grounds in MDD.

Cr, a significant marker of material metabolism, is the buffer that maintains the cell energy-dependent system by adjusting adenosine triphosphate (ATP) and adenosine diphosphate (Mountford et al., 2010). Cr concentrations have been considered to be relatively constant under normal conditions; thus, the ratio to Cr is widely used as an internal standard to scale other metabolites in traditional relative quantitative MRS. In fact, emerging evidence has found Cr concentrations in the brains of depressed patients are abnormal because of decreased mitochondrial ATP production and mitochondrial enzyme levels (Baxter et al., 1989; Gardner, 2003), which suggests that evaluating metabolism in depression with relative quantification might not be accurate, highlighting the necessity of absolute quantitative MRS. In this study, we quantified metabolite intensities by referencing internal water, which is the mature quantitative scheme preferred in clinical ^1^H-MRS. Additionally, the metabolic concentration of the hippocampus we measured was consistent with existing reports.

Although our study found that the dHDRS score (after 3 weeks) was an independent positive predictor, the data from the spectrum clearly showed a stronger prediction ability, which was reflected in the earlier time [2-week d(tCho)] and higher accuracy (3-week d(tCho), dNAA). NAA is a neuron internal marker that can reflect the functional status and integrity of neurons in the brain. Choline reflects cell membrane transport, which is generally assumed to play a key role in energy metabolism and myelination and has been proposed as a marker in pathological membrane renewal and cell membrane transport (Moffett et al., 2007; Oz et al., 2014). Patients who showed a > 50% reduction in HAMD scores after 6 weeks had higher levels of NAA or Cho and earlier clinical improvement, indicating that an increase in NAA or Cho is associated with treatment response. The differences in tCho and NAA between the RD group and the n-RD group, as well as their excellent predictive abilities, also indicated that the integrity of neurons, energy metabolism and myelination might be treatment targets for refractory depression in the future. In addition, compared with the rating scale of the neuropsychiatric disease evaluation system (HDRS), objective and sensitive brain material metabolism indicators may provide strong supplementary evidence.

Nevertheless, several limitations of this study should be noted. Firstly, refractory depression was defined as having no response to treatment with two antidepressants for 6–8 weeks. Although it is a commonly held view by psychiatrists, the optimal duration for a standard course of treatment has not been fully defined, and the definition of non-response is not completely clear. Therefore, the standard for the diagnosis of refractory depression may not be completely accurate. In addition, it is well demonstrated that glutamate and glutamine at 3.0T MRS are difficult to resolve and that the measurement of glutamate is likely to be affected by glutamine, albeit to a small degree. Thus, we took glutamate and glutamine as the whole Glx into consideration, but the change in glutamate could not be accurately estimated, which possibly affect the reliability. Poor test-retest reliability and difficult measurement of glutamate possibly result in poor correlation between Glx concentration and clinical improvement. We cannot rule out the effect that the sample size is limited due to difficulties in collecting clinical cases, and the need for multiple follow-ups limits the statistical ability to detect other findings. Moreover, due to the complexity of the pathogenesis of depression, a more sensitive and more specific logistic model and ROC curve should be generated, which requires further exploration. Despite these limitations, this study provides a way to predict the efficacy of antidepressants at an early stage with improved reliability. Once validated, biomarkers and the clinical assessment of patients with major depression could support psychiatrists’ diagnostic and treatment decisions and could increase the rationality of treatment.

## Conclusion

We developed a robust model to predict antidepressant responses based on short-term treatment changes, which may provide early guidance for adjusting treatment regimens for depression and serve as a checkpoint upon which the eventual outcome of conventional treatments can be predicted, reducing the time and resources wasted on ineffective treatment.

## Data availability statement

The data that support the findings of this study are available from Zhenjiang Mental Health Center but restrictions apply to the availability of these data, which were used only under license for the current study, and so are not publicly available. Requests to access the datasets should be directed to Yuefeng Li, jiangdalyf2009@126.com.

## Ethics statement

The studies involving human participants were reviewed and approved by the Ethics Committee of Zhenjiang Mental Health Center. The patients/participants provided their written informed consent to participate in this study.

## Author contributions

RW, YS, and AP made a substantial contribution to the concept and design, analysis, and interpretation of data. RW and YS drafted the article and processed MRI data. AP revised the article critically for important intellectual content. RD organized the study and supported the data analysis. All authors were critically involved in the theoretical discussion and performing of the experiments and read and approved the final version of the manuscript.

## Footnotes

1
www.fmrib.ox.ac.uk/fsl

2
https://pubchem.ncbi.nlm.nih.gov/compound/Water

## Abbreviations

AUC: area under curve
tCho: total choline (phosphocholine and glycerophosphocholine)
tCr: total creatine (creatine and phosphocreatine)
CSF: cerebrospinal fluid
PRESS: point resolved spectroscopy
FC: functional connectivity
FOV: field of view
FWHM: full width at half maximum
CRLBs: Cramér-Rao lower bounds
GLM: general linear model
Glx: glutamate and glutamine
GM: gray matter
HC: healthy control
HDRS-_17_: Hamilton Depression Rating Scale-_17_
Ins: myo-inositol
MRS: magnetic resonance spectroscopy
MDD: major depression disorder
NAA: N-acetyl -aspartate
n-RD: non-refractory depression
RD: refractory depression
ROC: receiver operating characteristic
ROI: region of interest
SNR: signal-to-noise ratio
WM: white matter.
