# Supplementary material for: Quantitative magnetic resonance spectroscopy of depression: The value of short-term metabolite changes in predicting treatment response
Source: Front Neurosci. 2022 Nov 29;16:1025882. doi: 10.3389/fnins.2022.1025882 (PMC9746341; doi:10.3389/fnins.2022.1025882)
Supplement: Supplementary file 1 [file Table_1.docx]

| **Table S1** The absolute concentration of metabolites in hippocampus from week0 to week6 | | | | | | | | | | |
| --- | --- | --- | --- | --- | --- | --- | --- | --- | --- | --- |
|  | | Week0 | Week1 | Week2 | Week3 | Week4 | Week5 | Week6 | F, df | *P* for trend |
| NAA | RD | 9.89±1.38 | 9.91±1.18 | 10.03±1.26**^b^** | 10.78±1.34**^a, b^** | 11.28±1.34 | 11.39±1.29 | 11.58±1.30 | 47.32, 1 | <0.001 |
|  | n-RD | 9.92±1.19 | 10.02±1.25 | 11.40±1.17**^a^** | 12.08±1.12**^a^** | 12.24±1.10 | 12.35±1.13 | 12.41±1.18 | 181.9, 1 | <0.001 |
|  | HC | 12.78±0.68 | 12.63±0.75 | 12.62±0.66 | 12.68±0.61 | 12.58±0.67 | 12.74±0.65 | 12.72±0.63 | <0.001, 1 | >0.999 |
| tCr | RD | 7.89±1.05 | 7.85±1.02 | 7.89±1.09 | 7.95±1.21 | 7.92±1.18 | 8.04±1.11 | 8.11±1.16 | 0.85, 1 | 0.358 |
|  | n-RD | 7.90±1.01 | 7.87±1.11 | 7.82±1.13 | 7.78±1.03 | 7.90±1.03 | 7.96±1.09 | 7.87±1.10 | 0.04, 1 | 0.848 |
|  | HC | 8.23±0.78 | 8.34±0.75 | 8.28±0.66 | 8.38±0.67 | 8.46±0.74 | 8.32±0.75 | 8.27±0.73 | 0.09, 1 | 0.763 |
| tCho | RD | 1.14±0.28 | 1.22±0.31 | 1.52±0.34**^a, b^** | 1.62±0.37**^a, b^** | 1.69±0.41 | 1.75±0.43 | 1.84±0.38 | 77.97, 1 | <0.001 |
|  | n-RD | 1.18±0.37 | 1.28±0.27 | 1.78±0.33**^a^** | 2.06±0.27**^a^** | 2.10±0.36 | 2.15±0.37 | 2.20±0.32 | 353.3, 1 | <0.001 |
|  | HC | 2.15±0.27 | 2.23±0.32 | 2.14±0.32 | 2.3±0.28 | 2.22±0.3 | 2.26±0.29 | 2.33±0.3 | 3.728, 1 | 0.056 |
| Glx | RD | 9.83±1.59 | 9.97±1.62 | 10.24±1.66 | 10.81±1.51**^a, b^** | 11.31±1.46 | 11.85±1.54 | 12.38±1.61 | 58.57, 1 | <0.001 |
|  | n-RD | 9.84±1.63 | 10.08±1.64 | 10.37±1.53 | 12.02±1.65**^a^** | 12.43±1.51 | 12.72±1.61 | 13.07±1.55 | 168.1, 1 | <0.001 |
|  | HC | 13.38±1.23 | 13.42±1.12 | 13.27±1.26 | 13.53±1.09 | 13.46±1.27 | 13.44±1.14 | 13.57±1.03 | 0.34,1 | 0.563 |
| Ins | RD | 5.55±0.71 | 5.62±0.78 | 5.72±0.83 | 5.65±0.85 | 5.74±0.73 | 5.83±0.82 | 5.78±0.79 | 1.91, 1 | 0.169 |
|  | n-RD | 5.61±0.92 | 5.83±0.89 | 5.74±0.91 | 5.85±0.87 | 5.78±0.82 | 5.86±0.77 | 5.81±0.82 | 0.97, 1 | 0.325 |
|  | HC | 6.7±0.58 | 6.65±0.64 | 6.78±0.66 | 6.8±0.56 | 6.61±0.57 | 6.58±0.62 | 6.64±0.58 | 0.47, 1 | 0.493 |

Note: RD, refractory depression; n-RD, non-refractory depression; NAA, N-acetyl-aspartate; tCho, phosphocholine and glycerophosphocholine; Glx, glutamine and glutamate; tCr, creatine and phosphocreatine; Ins, myo-inositol; df, degree of freedom; Data are shown as mean ± SD; The concentration of metabolites is in unit of mmol/kg; a, compared with week0, p<0.05; b, compared with n-RD, p<0.05. Statistical test, trend test.
