# Supplementary material for: Quantitative magnetic resonance spectroscopy of depression: The value of short-term metabolite changes in predicting treatment response
Source: Front Neurosci. 2022 Nov 29;16:1025882. doi: 10.3389/fnins.2022.1025882 (PMC9746341; doi:10.3389/fnins.2022.1025882)
Supplement: Supplementary file 2 [file Table_2.docx]

| Table S2 The diagnostic efficacy of metabolites predictor in validation set | | | | |
| --- | --- | --- | --- | --- |
|  | d(tCho)_week0-2_ | d(tCho)_week0-3_ | dNAA_week0-3_ | d(tCho)_week0-3_-dNAA_week0-3_ |
| Sensitivity | 0.636 | 0.727 | 0.727 | 0.818 |
| Specificity | 0.714 | 0.771 | 0.714 | 0.886 |
| AUC | 0.708 | 0.785 | 0.722 | 0.837 |
| 95%CI | 0.535-0.802 | 0.674-0.913 | 0.642-0.833 | 0.725-0.916 |
| *p* | 0.015 | 0.004 | 0.001 | 0.009 |

Note: d(tCho)_week0-2_, differentiated tCho (phosphocholine and glycerophosphocholine) after two weeks of treatment; d(tCho)_week0-3_, differentiated tCho after three weeks of treatment; dNAA_week0-3_, differentiated NAA (N-acetyl-aspartate) after three weeks of treatment; d(tCho)_week0-3_-dNAA_week0-3_, the combined detection of dNAA and d(tCho) after three weeks of treatment; AUC, area under curve; CI, confidence interval.
