# Supplementary material for: Quantitative magnetic resonance spectroscopy of depression: The value of short-term metabolite changes in predicting treatment response
Source: Front Neurosci. 2022 Nov 29;16:1025882. doi: 10.3389/fnins.2022.1025882 (PMC9746341; doi:10.3389/fnins.2022.1025882)
Supplement: Supplementary file 3 [file Table_3.docx]

| **Table S3** The spectral quality indices report | | | | | | | | | | |
| --- | --- | --- | --- | --- | --- | --- | --- | --- | --- | --- |
| Spectral quality |  | Week0 | Week1 | Week2 | Week3 | Week4 | Week5 | Week6 | F, df | P value |
| SNR | RD | 19.2±2.1 | 18.7±2.0 | 19.3±2.4 | 19.0±2.2 | 18.7±2.3 | 19.6±2.4 | 19.8±2.5 | 0.89, 6 | 0.502 |
|  | n-RD | 19.4±1.8 | 19.1±2.2 | 19.4±2.2 | 18.7±2.4 | 18.8±2.2 | 19.3±2.1 | 19.4±2.4 | 0.76, 6 | 0.600 |
| FWHM in Hz | RD | 7.8±1.5 | 7.2±1.9 | 7.4±2.1 | 7.1±1.7 | 8.2±1.8 | 7.3±1.6 | 7.6±1.5 | 1.27, 6 | 0.273 |
|  | n-RD | 7.7±1.7 | 7.4±2.0 | 7.2±2.2 | 6.9±1.5 | 7.9±1.6 | 7.5±1.8 | 7.7±1.7 | 1.47, 6 | 0.190 |
| CRLB (%) | | | | | | | | | | |
| NAA | RD | 2.2±0.7 | 2.4±0.6 | 2.6±0.7 | 2.3±0.7 | 2.7±0.8 | 2.6±0.7 | 2.5±0.8 | 1.64, 6 | 0.140 |
|  | n-RD | 2.3±0.6 | 2.5±0.8 | 2.5±0.8 | 2.4±0.6 | 2.6±0.7 | 2.4±0.6 | 2.7±0.7 | 1.56, 6 | 0.160 |
| Cr | RD | 2.2±0.8 | 1.9±0.7 | 2.2±0.8 | 2.3±0.7 | 2.5±0.8 | 2.3±0.9 | 2.4±0.8 | 1.51, 6 | 0.176 |
|  | n-RD | 2.1±0.9 | 2.0±0.8 | 2.3±0.8 | 2.2±0.9 | 2.3±0.7 | 2.2±0.8 | 2.5±0.7 | 1.63, 6 | 0.138 |
| Cho | RD | 5.8±2.3 | 6.1±2.6 | 6.4±2.5 | 5.8±2.3 | 6.8±2.1 | 6.9±2.2 | 5.9±2.0 | 1.07, 6 | 0.384 |
|  | n-RD | 5.7±2.5 | 5.9±2.8 | 6.3±2.4 | 6.0±2.4 | 6.6±2.0 | 6.8±2.4 | 6.0±2.1 | 1.14, 6 | 0.339 |
| Glx | RD | 8.6±3.0 | 9.5±2.8 | 8.9±2.8 | 9.6±2.6 | 9.3±2.7 | 8.9±2.9 | 10.2±2.8 | 0.97, 6 | 0.451 |
|  | n-RD | 8.6±2.7 | 9.4±2.9 | 9.1±2.7 | 9.7±2.9 | 9.0±3.0 | 9.1±2.8 | 10.0±2.9 | 1.11, 6 | 0.357 |
| Ins | RD | 4.7±2.2 | 5.3±2.4 | 5.2±2.2 | 4.8±2.2 | 6.0±1.9 | 5.7±2.0 | 4.9±2.1 | 1.31, 6 | 0.255 |
|  | n-RD | 4.8±2.1 | 5.2±2.4 | 5.1±2.4 | 4.9±2.3 | 5.8±2.1 | 5.7±1.9 | 4.8±2.3 | 1.43, 6 | 0.205 |
| Note：Data are shown as mean ± SD; CRLB, Cramer Rao Lower Bounds; FWHM, Full width at half maximum; SNR Signal to noise ratio; Data with SNR, linewidth (FWHM), and CRLB of metabolites calculated with Jmrui show high spectral quality of groups. The spectral quality show no significant difference between RD and n-RD and longitudinally; Statistical test, ANOVA. | | | | | | | | | | |
