# Supplementary material for: Quantitative magnetic resonance spectroscopy of depression: The value of short-term metabolite changes in predicting treatment response
Source: Front Neurosci. 2022 Nov 29;16:1025882. doi: 10.3389/fnins.2022.1025882 (PMC9746341; doi:10.3389/fnins.2022.1025882)
Supplement: Supplementary file 4 [file Table_4.docx]

| **Table S4** The voxel tissue fraction report | | | | | | | | | | |
| --- | --- | --- | --- | --- | --- | --- | --- | --- | --- | --- |
| Voxel tissue fraction |  | Week0 | Week1 | Week2 | Week3 | Week4 | Week5 | Week6 | F, df | P value |
| GM (%) | RD | 65.74±5.82 | 68.23±5.86 | 66.37±5.74 | 69.46±5.71 | 67.35±5.74 | 65.93±5.84 | 68.85±5.67 | 1.69, 6 | 0.125 |
|  | n-RD | 65.63±5.71 | 67.84±5.92 | 66.84±5.78 | 68.87±5.68 | 68.04±5.84 | 66.28±5.78 | 68.52±5.73 | 1.78, 6 | 0.102 |
| WM (%) | RD | 31.78±5.64 | 29.39±5.62 | 31.19±5.78 | 28.07±5.82 | 30.3±5.77 | 31.65±5.84 | 28.67±5.74 | 1.72, 6 | 0.119 |
|  | n-RD | 31.91±5.83 | 29.81±5.74 | 30.72±5.63 | 28.68±5.72 | 29.58±5.63 | 31.32±5.67 | 29.03±5.68 | 1.82, 6 | 0.095 |
| CSF (%) | RD | 2.48±0.22 | 2.38±0.19 | 2.44±0.21 | 2.47±0.24 | 2.35±0.18 | 2.42±0.23 | 2.48±0.22 | 1.49, 6 | 0.185 |
|  | n-RD | 2.46±0.21 | 2.35±0.23 | 2.44±0.22 | 2.45±0.20 | 2.38±0.21 | 2.40±0.18 | 2.45±0.21 | 1.67, 6 | 0.128 |
| Note：Data are shown as mean ± SD; GM, Grey matter (%); WM, White matter (%); CSF, Cerebrospinal fluid (%); df, degree of freedom; The voxel tissue fraction show no significant difference between RD and n-RD and longitudinally. Statistical test, ANOVA. | | | | | | | | | | |
